# Auto-sumoylation of the Ubc9 E2 SUMO-conjugating Enzyme Extends Cellular Lifespan

**DOI:** 10.21203/rs.3.rs-4016606/v1

**Published:** 2024-03-21

**Authors:** Hong-Yeoul Ryu, Dong-Won Jeong, Seung Yeon Kim, Seok-Won Jeoung, Dejian Zhao, James Knight, TuKiet Lam, Jong Hwa Jin, Hyun-Shik Lee, Mark Hochstrasser

**Affiliations:** Kyungpook National University; Kyungpook National University; Kyungpook National University; Kyungpook National University; Yale University; Yale University; Keck MS & Proteomics Resource, Yale School of Medicine; Osong Medical Innovation Foundation; Kyungpook National University; Yale University

**Keywords:** SUMO, Ubc9, lifespan, energy metabolism, calorie restriction

## Abstract

Calorie restriction (CR) provides anti-aging benefits through diverse processes, such as reduced metabolism and growth and increased mitochondrial activity. Although controversy still exists regarding CR-mediated lifespan effects, many researchers are seeking interventions that mimic the effects of CR. Yeast has proven to be a useful model system for aging studies, including CR effects. We report here that yeast adapted through *in vitro* evolution to the severe cellular stress caused by loss of the Ulp2 SUMO-specific protease exhibit both enhanced growth rates and replicative lifespan, and they have altered gene expression profiles similar to those observed in CR. Notably, in certain evolved *ulp2Δ* lines, a dramatic increase in the auto-sumoylation of Ubc9 E2 SUMO-conjugating enzyme results in altered regulation of multiple targets involved in energy metabolism and translation at both transcriptional and post-translational levels. This increase is essential for the survival of aged cells and CR-mediated lifespan extension. Thus, we suggest that high Ubc9 auto-sumoylation exerts potent anti-aging effects by promoting efficient energy metabolism-driven improvements in cell replication abilities. This potential could be therapeutically explored for the development of novel CR-mimetic strategies.

## Introduction

All organisms transmit heritable characteristics across successive generations in response to environmental changes, including climate fluctuations, habitat modifications, and food scarcity^1^. At the cellular level, both external and internal environmental changes, such as oxidative stress, nutrient deprivation, or genotoxic stress, typically trigger a range of immediate and long-term adaptive responses to counterbalance the stress stimuli^2,3^. During acute stress, initial cellular responses (such as in metabolism, protein homeostasis, or enzyme activities) work to swiftly reestablish equilibrium and sustain viability. When these protective responses are not sufficient to mitigate the effects of stress, secondary adaptive mechanisms are implemented that primarily include genetic changes to resist stress. One useful approach for studying such adaptive mechanisms is *in vitro* laboratory evolution, which includes the artificial induction of cellular or genetic alterations under specific growth conditions that are difficult to produce in nature and offers insights into molecular adaptations in response to environmental change^4^.

The small ubiquitin-like modifier (SUMO) protein is a crucial regulator of cell homeostasis, especially during environmental stress^5^. In humans, five genes encode SUMO paralogs^6,7^, while the budding yeast *Saccharomyces cerevisiae* expresses a single SUMO ortholog, Smt3, which shares 48% identity with human SUM01^6^. SUMO is initially synthesized as a C-terminally extended precursor and later processed into its mature form by SUMO-specific proteases (Ulp1 in *S. cerevisiae)*. The mature SUMO is then conjugated to lysine (K) side chains of target proteins via an enzyme cascade similar to the ubiquitin conjugation process^8^. Initially, a heterodimeric E1 SUMO-activating enzyme (Aos1/Uba2 in *S. cerevisiae)* forms a thioester linkage between Uba2 and the carboxy terminus of SUMO. The SUMO moiety is then transferred to the active-site cysteine of the Ubc9 E2 SUMO-conjugating enzyme. SUMO can form polymeric chains on substrates that are disassembled by site-specific SUMO proteases. Humans have nine known SUMO proteases, while *S. cerevisiae*has two (Ulp1 and Ulp2)^9^.

The SUMO stress response involves rapid changes in SUMO conjugates in response to various stressors and is not yet fully understood. In *S. cerevisiae*, the Siz1 E3 ligase and Ulp2 SUMO protease play crucial roles in the SUMO stress response^10^. The primary activity of Ulp2 is as a SUMO chain editor^11^. Loss of the *ULP2* gene leads to many severe cellular defects, including defects in growth, DNA and mitotic checkpoint recovery, chromosome stability, and meiosis^12^.

SUMO system dysregulation after Ulp2 protease loss triggers both short-term and long-term adaptive mechanisms, depending on the continuous culture duration. Short-term adaptation includes the development of a specific multi-chromosome aneuploidy and significant changes in the transcription of ribosomal genes, which promotes compensatory mechanisms for rapid adaptation to Ulp2 loss^13,14^. Because aneuploidy is typically detrimental to cellular fitness^15^, long-term adaptation restores euploidy and growth, adjusts transcription, and often leads to countervailing mutations in SUMO-conjugation enzymes^14^. This stepwise utilization of adaptive mechanisms is relevant to the robust adaptive fitness gains observed in *ulp2Δ* cells following extended serial culturing.

Aging is a process characterized by the gradual accumulation of molecular, cellular, and organ damage during sexual maturity that ultimately increases illness and mortality susceptibility^16^. *S. cerevisiae serves* as a valuable model organism for studying the aging process using two distinct approaches based on either replicative lifespan (RLS) or chronological lifespan (CLS)^17^. In RLS, the number of mitotic cycles completed by an originating cell before cell senescence is analyzed and used to help understand lifespan determinants of proliferating cells, such as stem cells. In contrast, CLS focuses on understanding the aging process in nondividing yeast cells over time, which simulates postmitotic cells in multicellular organisms. Lifespan studies on yeast have revealed diverse conserved genetic pathways that influence aging, including the sirtuin (Sir2) histone deacetylase (HDAC)-mediated maintenance of chromatin stability and the target of rapamycin (TOR) signaling pathway for nutrient sensing and growth control^18^.

Calorie restriction (CR), which promotes longevity in a wide range of species, including mammals, is closely linked to both the Sir2 and TOR pathways. While Sir2 activity is enhanced by the altered NAD/NADH ratio under starvation, CR also suppresses the TOR pathway, resulting in increased mitochondrial respiration and reduced oxidative damage^18^. Our understanding of these mechanisms remains incomplete, in part due to conflicting experimental results on questions such as whether a prolonged lifespan is ensured by respiratory chain dysfunction in *Caenorhabditis elegans* and the Sir2-independent CR pathway in *S. cerevisiae*^19,20^. At the same time, the SUMO pathway regulates diverse cellular processes involved in aging, including telomere function, autophagy, oxidative stress responses, and growth factor signaling^21^. Thus, a better understanding is needed of the mechanisms connecting sumoylation to the regulation of energy metabolism and other biological events in senescence.

In our previous work, we had found that different yeast lines that evolved *in vitro* from the same founding nascent *ulp2Δ* colony followed distinct evolutionary paths to restore euploidy, robust growth, and stress resistance^14^. Most commonly, mutations in SUMO conjugation pathway enzymes were identified and these correlated with a strong drop in high molecular weight (HMW) polysumoylated proteins. In several of the evolved lines, however, SUMO-conjugate levels remained abnormally high, and no additional mutations to SUMO pathway enzymes were identified.

We have therefore undertaken genome-wide gene expression studies to investigate how such *in vitro* evolved *ulp2Δ* cell lines could nevertheless overcome the defects caused by the loss of the Ulp2 protease. Genes related to energy metabolism and protein translation underwent clear changes, with the former being upregulated and the latter being downregulated in the evolved SUMO conjugate-replete *ulp2Δ* cells. These changes correlated with increases in growth rate and stress resistance and RLS extension depended on mitochondrial respiration.

Strikingly, these evolved *ulp2Δ* cells experienced strongly increased levels of auto-sumoylated Ubc9 enzyme. Auto-sumoylation of Ubc9 specifically altered expression and SUMO conjugation of proteins involved in energy metabolism and translation. Interestingly, preventing Ubc9 auto-sumoylation blocked the marked accumulation of SUMO conjugates normally seen in replicatively aged WT cells and also eliminated the prolonged RLS induced by CR. Ubc9 auto-sumoylation was previously shown to alter the substrate preferences of the SUMO conjugation pathway^22,23^. Our data suggest that enhanced Ubc9 auto-sumoylation adjusts energy metabolism via gene expression and protein sumoylation changes during aging. This remarkable metabolic malleability enhances replication capacity and the survival of aged cells and may mimic the effects of CR on lifespan extension.

## Results

### Gene expression pattern in ulp2Δ cells is altered after multiple generations

**Figure 1:**
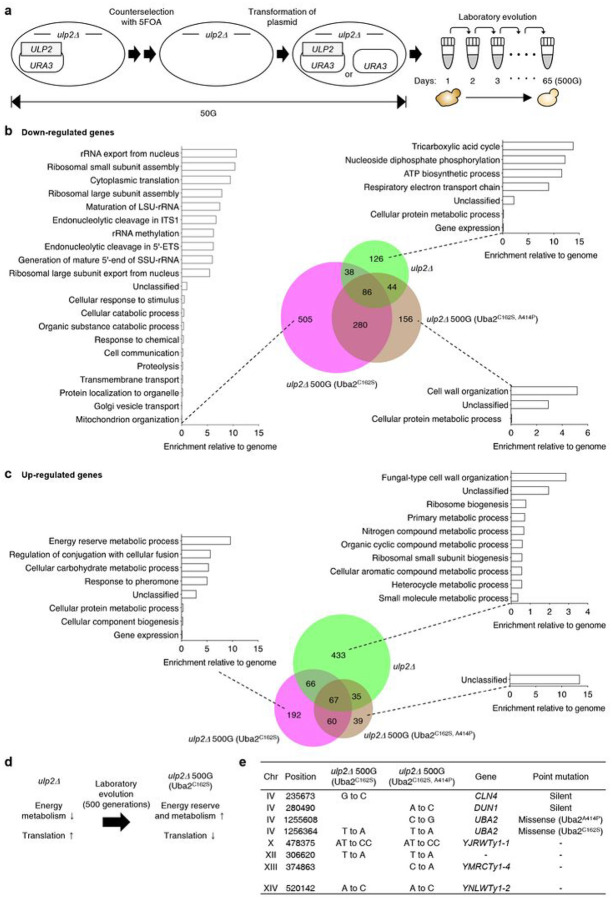
Changes in gene expression profiles in *ulp2Δ* cells during passaging. **a**, Scheme for the creation of nascent *ulp2Δ* cells and subsequent laboratory evolution steps. The *ulp2Δ* cells containing YCplac33-*ULP2*(MHY1379) were sequentially streaked on SD + FOA plates twice to evict the YCplac33-*ULP2* plasmid and then transformed with either YCplac33 orYCplac33-*ULP2*. Cells grew for ~50 generations (G) during these procedures. The cells were grown until saturation, then diluted 1:120 in fresh YPD (6.9 generations per dilution) for long-term passaging. This process was repeated daily for 65 days (~500G), which corrected the growth defects of *ulp2Δ* cells. **b,c**, Venn diagram and Gene Ontology (GO) enrichment analysis of genes with significantly decreased (<2-fold, **b)** or increased (>2-fold, **c)** expression in nascent (low passage) *ulp2D, ulp2D* 500G (Uba2^C162S^), and *ulp2D* 500G (Uba2^C162S, A414P^), compared to WT strain MHY1379 (*ulp2Δ* + YCplac33-*ULP2*). Bars indicate the fold-enrichment of each GO biological process in PANTHER (http://pantherdb.org/). The genes used for GO analysis are listed in Supplementary Data 1. **d**, Schematic summary of the expression changes in *ulp2D* 500G (Uba2^C162S^) compared to nascent *ulp2Δ* cells depicted in **b** and **c. e**, Mutations in *ulp2D* 500G (Uba2^C162S^) and *ulp2D* 500G (Uba2^C162S, A414P^). Missense or silent represents alteration of amino acids by point mutation or not, respectively.

We recently reported that growth and cell cycle defects in *ulp2Δ* cells were restored after ~ 500 generations (G) of continuous culture with repeated cycles of dilution and outgrowth in fresh medium^13,14^ (Fig. 1a). Missense mutations in essential SUMO conjugation pathway components (Uba2, Aos1, or Ubc9) were observed in all of the laboratory evolved *ulp2Δ* cells. In one set of 10 lines evolved in parallel, a single Uba2 cysteine-to-serine point mutation at position 162 (C162S) was observed in all the lines and is presumed to have arisen in the founder colony. However, this did not suppress the growth and cell-cycle impairments and had only a mild suppressive effect on the accumulation of HMW polySUMO conjugates in nascent *ulp2Δ* cells. In contrast, the other mutants had additional mutations in either Uba2 or Aos1, including *uba2*^C162S, A414P^ (alanine 414 to proline), and these additional mutations strongly reduced HMW polySUMO conjugate accumulation in the evolved *ulp2Δ* strains, which mitigated the defects in growth and cell-cycle progression^14^. Thus, the mechanism of cell adaptation to Ulp2 loss in evolved *ulp2Δ* cells with only the Uba2-C162S mutation, has remained unclear.

To begin analyzing potential adaptive mechanisms in the *ulp2Δ* 500G (Uba2^C162S^) strain, gene expression profiles were analyzed by genomic RNA sequencing (RNA-seq; Fig. 1b, c **and Supplementary Data 1**). Levels of many transcripts were substantially different (two-fold or more) in the indicated strains compared to expression in the WT. In nascent *ulp2Δ* cells, the number of genes with increased transcript levels (601) (Fig. 1c) was more than double that of genes with reduced transcript levels (294) (Fig. 1b). Interestingly, the overall changes in transcript levels were much more muted in the two evolved *ulp2Δ* strains tested; specifically, the ratio of the total number of significantly up- and down-regulated genes in nascent *ulp2Δ* cells was more than double that of the two evolved strains (*ulp2Δ* 500G (Uba2^C162S^; 909 vs. 385) and *ulp2Δ* 500G (uba2^C162S, A414P^; 566 vs. 201)). This indicates a general return to a WT gene expression balance in the evolved *ulp2Δ* strains despite the irreversible loss of Ulp2.

Energy metabolism genes, including tricarboxylic acid (TCA) cycle, ATP biosynthesis, and respiratory electron transport chain-related genes, were specifically down-regulated in nascent *ulp2Δ*, whereas ribosomal proteins and SUMO-regulated ribosome biogenesis factors^24^ were heavily enriched among the genes with a strong increase in expression. In direct contrast to this pattern, genes required for translation, including those involved in rRNA maturation, ribosome assembly and nuclear export, and translation factors, were down-regulated evolved *ulp2Δ* 500G (Uba2^C162S^) cells, while genes for energy reserve and cellular carbohydrate metabolic processes were upregulated in this line. In particular, a significant increase was seen in the mRNA levels for enzymes involved in converting glucose to glycogen and, conversely, for enzymes that catalyze catabolic reactions that break down carbohydrates, including hexokinase (Hxk1) in glycolysis, citrate synthase (Cit2) in the TCA cycle, and glycogen debranching enzyme (Gdb1) required for mobilizing glucose reserves from glycogen deposits^25–27^. These data implied that both the energy storage and consumption processes were markedly activated in *ulp2Δ* 500G (Uba2^C162S^) cells (Fig. 1c **and Supplementary Data 1**). Notably, any significant feature was not observed in the Gene Ontology (GO) analysis for the *ulp2Δ* 500G (Uba2^C162S, A414P^) line. In short, our RNA-seq data revealed that the decrease in enzymes for energy metabolism and increase in translation capacity seen in nascent *ulp2Δ* cells were reversed in the *ulp2Δ* 500G (Uba2^C162S^) strain (Fig. 1d) but not in the *ulp2Δ* 500G (Uba2^C162S, A414P^) line, suggesting distinct long-term adaptation mechanisms.

We previously reported several adaptive mutations that provide a selective advantage to evolved *ulp2Δ* cells^14^. We searched for other potential gene mutations in *ulp2Δ* 500G (Uba2^C162S^) and *ulp2Δ* 500G (Uba2^C162S, A414P^) by genome sequencing but did not find any alterations that would lead to changes in protein sequence except for the noted Uba2 mutations (Fig. 1e). To test whether the enhanced expression of the energy metabolism genes observed in *ulp2Δ* 500G (Uba2^C162S^) could influence the slow growth of nascent *ulp2Δ* cells, we transformed the WT strain (*ulp2Δ + pULP2*) with each of 33 different library plasmids containing identified energy metabolism genes and then analyzed cell growth via serial dilution analysis after evicting the *ULP2* cover plasmid **(Extended Data** Fig. 1). None of the tested high-copy genes by themselves suppressed the growth impairment in the *ulp2Δ* cells. Altogether, these results imply that the growth recovery of *ulp2Δ* 500G (Uba2^C162S^) results from an as yet unidentified (possibly nongenetic) adaptive mechanism that likely requires altered expression of more than a single metabolic enzyme.

### Growth rate and RLS are increased in high-passage ulp2Δ cells

**Figure 2:**
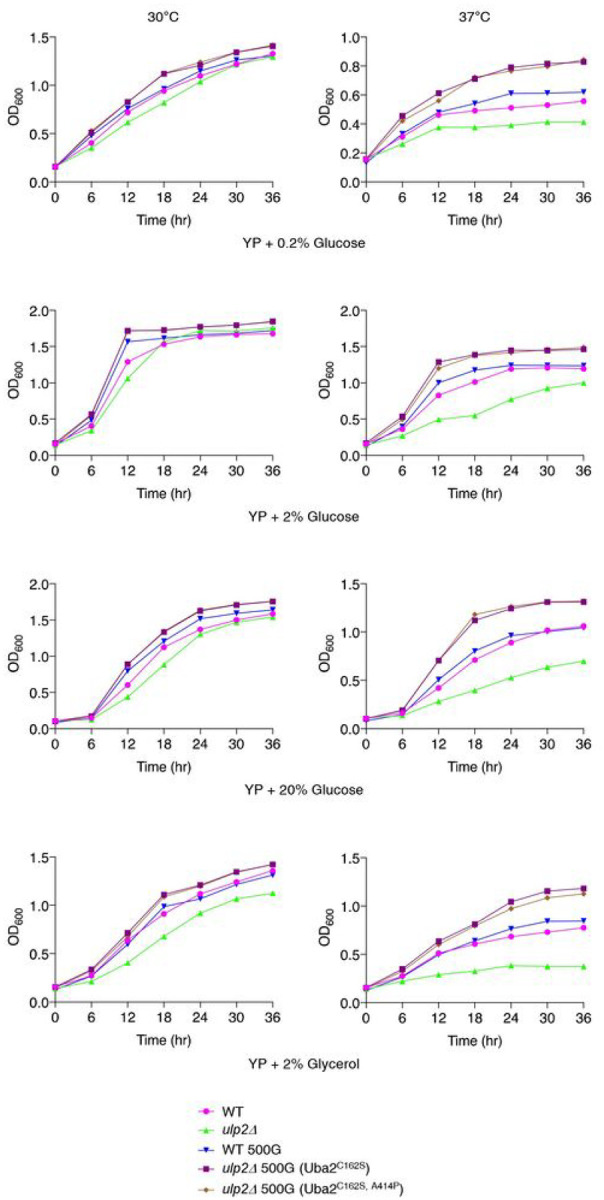
Growth rates of high-passage *ulp2Δ* cells exceed WT rates. Cells of the indicated strains pregrown in yeast extract-peptone-dextrose (YPD; 2% glucose) at 30°C were adjusted to an optical density (OD)_600_ value of 0.1, then cultured in YP with 0.2%, 2%, or 20% glucose or 2% glycerol at either 30°C or 37°C. Growth curves were derived from OD_600_ measurements every 6 h for 36 h.

Metabolic energy balance is important for cell growth and function; its malfunction is implicated in many complex diseases and aging^19^. To determine whether the elevated energy metabolism transcripts affect the growth rate of *ulp2Δ* 500G (Uba2^C162S^), we analyzed the growth curves of the indicated cells at different temperatures and using different concentrations and sources of carbon (Fig. 2 **and Extended Data** Fig. 2). With optimal temperature and carbon source (30°C and 2% glucose), typical S-shaped growth curves were observed for both the low passage WT and WT 500G cells. By 12 h, all strains except low passage *ulp2Δ* cells appeared to have slowed their doubling rate (diaxic shift). Cell density at saturation was slightly decreased in nascent *ulp2Δ* cells but had increased relative to WT in the evolved *ulp2Δ* cells. When the culture conditions were changed to low (0.2%) or very high (20%) glucose concentrations, a high temperature (37°C), or a nonfermentable carbon source (2% glycerol), the time needed to reach saturation was delayed and the growth rate changes in the mutants were much greater than under optimal conditions. Remarkably, the two evolved *ulp2Δ* lines had a clear growth advantage relative to WT cells, especially at 37°C. These findings indicate that laboratory evolution can induce beneficial effects beyond the correction of vegetative growth defects in *ulp2Δ* cells.

**Figure 3:**
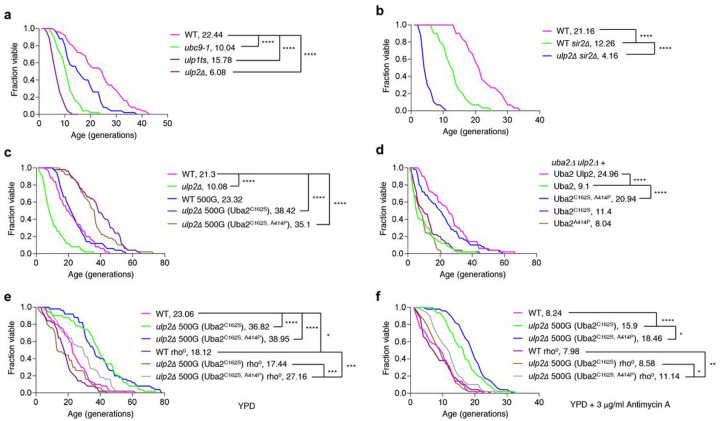
Replicated lifespan (RLS) is extended in evolved *ulp2Δ* cells. **a-f**, RLS analysis of indicated strains **(a-e)** and *uba2Δ ulp2Δ* cells with indicated plasmids **(f**). RLS was measured on yeast extract-peptone-dextrose (YPD) **(a-d, f)** and YPD supplemented with 3 jjg/mL Antimycin A **(e)**, respectively. Rho^0^ represents cells lacking mitochondrial DNA. The RLS means are shown in parentheses. Asterisks indicate statistically significant differences (*P < 0.05; **P < 0.01; *** *P* < 0.001; ****P < 0.0001; Wilcoxon Rank Sum Test).

It is commonly assumed that growth rate is negatively coupled with lifespan, but some evidence indicates a positive correlation^28,29^. The SUMO pathway has been linked with cellular senescence and the organismal aging process. Its substrates include several factors that control cellular senescence, such as p53, RB, and SIRT1^21^. Thus, we next examined the change in RLS caused by the genetic mutation or deletion of the main SUMO pathway proteins (Ubc9, Ulp1, and Ulp2; Fig. 3a). We observed that normal lifespan was significantly shortened in all the tested mutant strains (*ubc9-1*, *ulp1-ts*, and nascent *ulp2Δ*) suggesting that the dynamic regulation of SUMO conjugation and deconjugation is required for maintaining a normal lifespan.

Because Sir2 is a well-known factor that modulates RLS^30–32^, we subsequently investigated whether Sir2 affects RLS in the *ulp2Δ* strain (Fig. 3b). Consistent with previous results, RLS was decreased in *sir2Δ*, and the absence of Ulp2 further reduced *sir2Δ* lifespan; the latter result implies that Ulp2 plays a role in lifespan modulation separate from that of Sir2. In particular, this finding reveals a novel Sir2-independent pathway that sustains RLS by controlling sumoylation level.

Remarkably, lifespan was greatly extended in both evolved *ulp2Δ* strains, with RLSs as much as 80% above WT (Fig. 3c). As noted above, these same strains also exhibited higher growth rates relative to WT (Fig. 2). High rates of cell growth generally correlate with a shorter lifespan, but there are many exceptions. For instance, the loss of the global transcriptional regulator Sus1 or ubiquitin-specific protease Ubp10 simultaneously reduces growth and RLS^33,34^. Previously, we reported that several Uba2 double mutants, which included the C162S point mutation, suppressed the growth and cell-cycle defects seen in nascent *ulp2Δ* cells by reducing HMW SUMO-conjugated protein levels^14^. The C162S mutation by itself did not. To determine whether this reduction caused by Uba2 mutation affects RLS and growth, we analyzed the RLS and growth changes in *uba2Δ* and *ulp2Δ* strains expressing Uba2, uba2^C162S, A414P^, Uba2^C162S^, or Uba2^A414P^ (Fig. 3d **and Extended Data** Fig. 3). The expression of Uba2^C162S, A414P^ eliminated the shortened lifespan and proportionally rescued the growth defects in *ulp2Δ*; these effects were not seen with in *ulp2Δ* cells carrying the singly mutated Uba2^C162S^ or Uba2^A414P^. These findings suggest that Uba2 activity must be reduced to below a certain threshold, in this case requiring two Uba2 mutations, before RLS or growth impairment in *ulp2Δ* is rescued. The Uba2 double mutation may also be crucial for the RLS change in *ulp2Δ* 500G (Uba2^C162S, A414P^).

Taken together, the growth curve and RLS analyses showed that a normal limitation on cellular replication ability was overcome in both of the tested laboratory-evolved *ulp2Δ* strains, suggesting that adaptive mechanisms for overcoming Ulp2 loss might extend cellular lifespan.

### Extended RLS linked to altered energy metabolism in ulp2Δ 500G (Uba2^C162S^)

Based on the RNA-seq experiments (Fig. 1b, c) we next addressed the impact the apparent elevated energy metabolism had on the lifespan of *ulp2Δ* 500G (Uba2^C162S^) (Fig. 3e **and Extended Data** Fig. 4a). Mitochondrial DNA (mtDNA) is necessary for mitochondrial respiration and energy metabolism^35^, leading many researchers to investigate RLS in cells lacking mtDNA (rho^0^ cells) to determine whether mitochondrial respiration affects RLS. Previous results show that the impact of mtDNA loss on cell longevity depends on the yeast strain^36–38^.

We found that the RLS of WT MHY1379 cells which had been rendered rho^0^ was decreased by about 20% compared to that of the WT, which is consistent with previous results^37^. Moreover, the extended RLS of *ulp2Δ* 500G (Uba2^C162S^) was abolished by loss of mtDNA, whereas *ulp2Δ* 500G (Uba2^C162S, A414P^) rho^0^ cells retained a significantly higher RLS, suggesting that mitochondrial function is a key element to prolonged RLS in *ulp2Δ* 500G (Uba2^C162S^) and that another pathway, perhaps SUMO regulation, is required for the extended lifespan of *ulp2Δ* 500G (Uba2^C162S, A414P^). The addition of antimycin A, which specifically blocks mitochondrial respiration and reduces RLS^37^, also more significantly impaired RLS in *ulp2Δ* 500G (Uba2^C162S^) than in *ulp2Δ* 500G (Uba2^C162S, A414P^ cells) (Fig. 3f **and Extended Data** Fig. 4b). Thus, our results suggest that extended RLS in *ulp2Δ* 500G (Uba2^C162S^) cells results from a shift in energy metabolism.

### Ubc9 auto-sumoylation is increased in ulp2Δ 500G (Uba2^C162S^)

**Figure 4:**
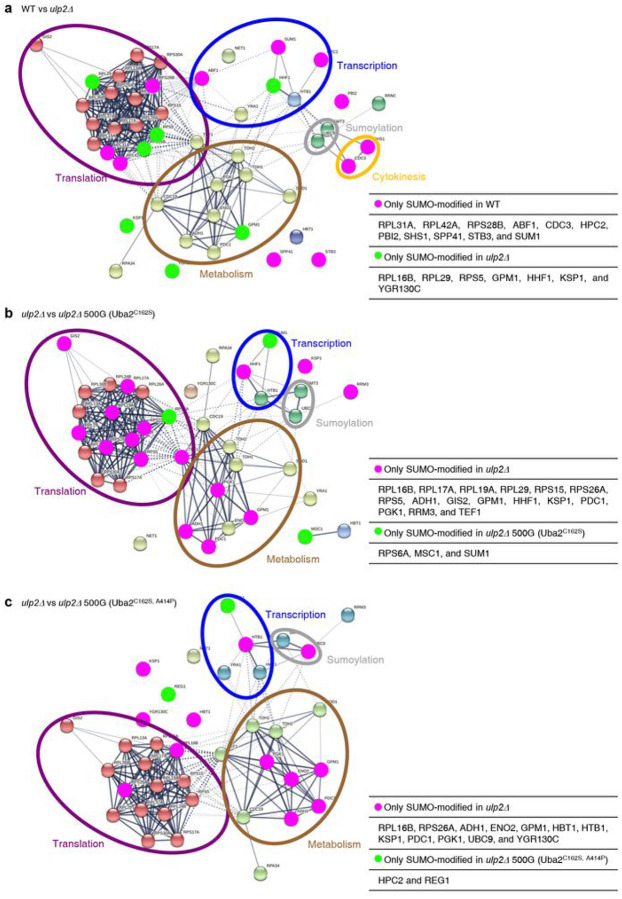
Analysis of SUMO-modified proteins in evolved *ulp2Δ* cells. **a-c**, Sumoylated proteins were purified under denaturing conditions from the wild-type (WT), *ulp2D, ulp2D* 500G (Uba2^C162S^), and *ulp2D* 500G (Uba2^C162S^) cells expressing 6His-FLAG–tagged SUMO and were identified via mass spectrometry. Sumoylated proteins were compared between the indicated groups **(a**, WT and *ulp2D*, **b**, *ulp2D and ulp2D* 500G (Uba2^C162S^); and **c**, *ulp2D and ulp2D* 500G (Uba2^C162S, A414P^)). The protein interaction network was constructed via STRING (https://string-db.org/). Each node and distance between the nodes indicate identified proteins and their relatedness, respectively. Magenta and green circles denote the SUMO-modified proteins in each strain only. Violet, blue, brown, gray, and yellow open circles indicate proteins involved in translation, transcription, metabolism, sumoylation, and cytokinesis, respectively.

Because the global profile of sumoylated proteins was altered in the evolved *ulp2Δ* strains **(Extended Data** Fig. 4c), we speculated that changes in SUMO substrates may contribute to cellular transcriptome changes relevant to RLS increase. We performed affinity purification in the evolved *ulp2Δ* strains expressing N-terminal 6His-FLAG (HF)-tagged Smt3 followed by mass spectrometry analyses to identify SUMO-modified proteins (Fig. 4, **Extended Data** Fig. 5, **and Supplementary Data. 2**). In agreement with previous findings^39,40^, HF-Smt3 was linked with multiple proteins involved in transcription, translation, metabolism, cytokinesis, and sumoylation in the WT (Fig. 4a). As expected, the initial Ulp2 loss led to broad changes in the SUMO-conjugate profile (Fig. 4a), which supports the conclusion that the Ulp2 SUMO protease has many target proteins required for diverse pathways, including transcription, the cell cycle, and ribosome biogenesis^9^. Both evolved *ulp2Δ* strains tended to deplete rather than increase SUMO-conjugated proteins; among them were proteins involved in energy metabolism and translation, including key enzymes need for glycolysis and gluconeogenesis (e.g., Adh1, Gpm1, Pdc1, Pgk1, and Eno2^41–43^), several ribosome subunits, translation control factors (Gis2 and Tef1)^44,45^, and Ksp1, a kinase involved in TOR signaling (Fig. 4b, c)^46^.

**Figure 5:**
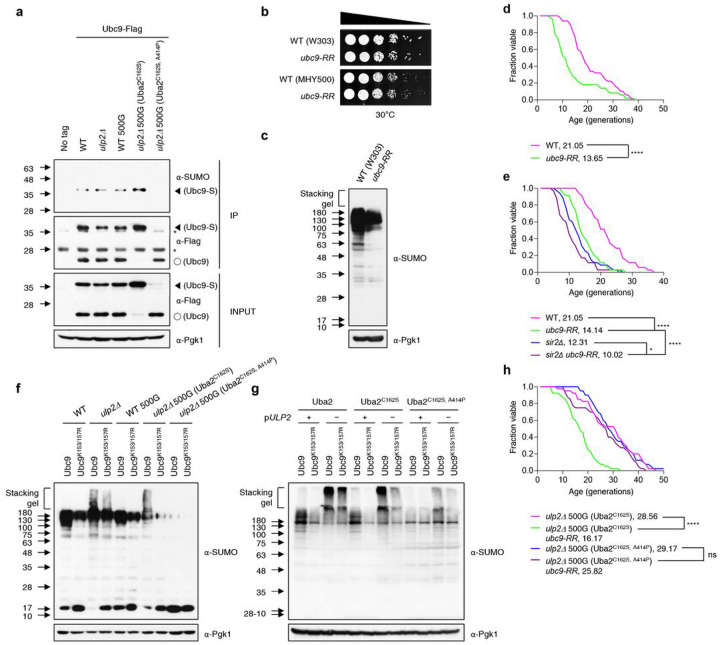
Auto-sumoylation of Ubc9 is enhanced in *ulp2D*500G (Uba2^C162S^) cells. **a**, Immunoprecipitation (IP) of Ubc9-FLAG with anti-FLAG agarose from denatured yeast extracts in the indicated strains expressing FLAG-tagged Ubc9 followed by immunoblot analysis with anti-SUMO or anti-FLAG antibodies. Anti-Pgk1 was used as a loading control for protein input. Arrowheads, open circles, and asterisks indicate sumoylated Ubc9, unsumoylated Ubc9, and nonspecific bands, respectively, **b**, Growth of the *ubc9-K153/157R (ubc9-RR)* mutants from two different strain backgrounds (W303 and MHY500). After spotting cells in five-fold serial dilutions, the YPD plates were incubated for 2 days at 30°C. **c,f,g**, Immunoblot assay of sumoylated proteins in extracts prepared from the indicated strains. The plus and minus symbols represent the presence and absence of YCplac33-*ULP2* in the indicated *uba2, ubc9*, and *ulp2* mutants, respectively. Anti-Pgk1 blotting was used to verify equal loading. The stacking gel (bracket) and molecular size standards are indicated. **d,e,h**, RLS analysis of indicated strains, as shown in Fig. 3a.

Among the proteins characterized by changes in SUMO modification in the evolved strains, we were especially intrigued by the large increase in SUMO-conjugated Ubc9 accompanied by a corresponding decrease in its unconjugated state in *ulp2Δ* 500G (Uba2^C162S^) cells (Fig. 5a). Conversely, SUMO-modified Ubc9 almost disappeared in the *ulp2Δ* 500G (Uba2^C162S, A414P^) strain, presumably due to the extremely low levels of SUMO conjugates. Previous work demonstrated that the sumoylation of human Ubc9-Lys14 (K14) inhibits its ability to sumoylate the well-known target RanGAP1 but promotes modification of another substrate, Sp100^22^. In *S. cerevisiae*, Ubc9 auto-sumoylation is also observed, in this case on two residues, K153 and K157; this negatively regulates septin sumoylation, which is required for maintaining normal cell morphology^23^. Thus, the unusually high levels of Ubc9 auto-sumoylation seen in the *in vitro* evolved *ulp2Δ* 500G (Uba2^C162S^) strain and potentially the reduced levels of such modification in *ulp2Δ* 500G (Uba2^C162S, A414P^) cells is predicted to rewire cellular SUMO target discrimination.

### Ubc9 auto-sumoylation increases RLS in a Sir2-independent manner

It was previously reported that Ubc9 auto-sumoylation was nearly abolished in a *ubc9-K153/157R (ubc9-RR)* mutant; this did not strongly affect vegetative growth or general SUMO conjugation but caused a severe reduction of global cellular sumoylation during meiosis^47^. We confirmed that cell growth did not differ between the WT and *ubc9-RR* strains in two different genetic backgrounds (W303 and MHY500; Fig. 5b); however, global SUMO conjugates were comparatively reduced in the *ubc9-RR* cells (Fig. 5c). Because the *ulp2Δ* 500G (Uba2^C162S^) strain displayed enhanced Ubc9 auto-sumoylation and extended RLS, we tested the effect of Ubc9 auto-sumoylation on RLS (Fig. 5d). The RLS of the *ubc9-RR* mutant was indeed shorter than that of WT cells, suggesting that Ubc9 auto-sumoylation is required for maintaining a normal lifespan. Moreover, the lifespan of a *sir2Δ ubc9-RR* double mutant was significantly shorter than that of either single mutant, indicating an additive effect on lifespan when these mutations are combined (Fig. 5e). As noted in Fig. 3b, Ulp2 loss shortens RLS in a Sir2-independent manner, and the results in Fig. 5e suggest that Ubc9 auto-sumoylation also contributes to a normal RLS through a Sir2-independent mechanism. Thus, our data indicate that the SUMO pathway contributes to yeast lifespan in a way unrelated to Sir2 function.

### Ubc9 auto-sumoylation is required for RLS extension in ulp2Δ 500G (Uba2^C162S^)

To assess the effect of Ubc9 auto-sumoylation on global sumoylation in *ulp2Δ* 500G (Uba2^C162S^) cells, we measured SUMO-conjugate levels by anti-SUMO immunoblotting of extracts from *ulp2Δ* 500G (Uba2^C162S^) cells also bearing the *ubc9-RR* mutant allele (Fig. 5f). The *ubc9-RR* allele led to a sharp decline in HMW SUMO conjugates in the *ulp2Δ* 500G (Uba2^C162S^) background, while a more moderate reduction was seen in nascent *ulp2Δ* cells, suggesting that Ubc9 auto-sumoylation is an important factor in the maintenance of cellular SUMO conjugates in evolved *ulp2Δ* (Uba2^C162S^) cells.

While Ubc9^K153/157R^ expression only weakly reduced polySUMO conjugate accumulation in *ulp2Δ* cells expressing WT Uba2 (Fig. 5g, lanes 3–4), Ubc9^K153/157R^ expression in the *ulp2Δ* strain expressing Uba2^C162S^ efficiently suppressed accumulation of the excess HMW polySUMO conjugates (Fig. 5g, lanes 7–8). This finding implies that the C162S mutation of Uba2 enhances suppression of the *ulp2Δ* defect by the auto-sumoylation-resistant ubc9^K153/157R^ protein. To determine whether Ubc9 auto-sumoylation influences long-lived *ulp2Δ* 500G (Uba2^C162S^) cells, we examined RLS in evolved *ulp2Δ* cells into which the *ubc9-RR* allele was introduced (as the only source of Ubc9) (Fig. 5h). The *ubc9-RR* allele dramatically reduced RLS in the *ulp2Δ* 500G (Uba2^C162S^) strain but not in *ulp2Δ* 500G (Uba2^C162S, A414P^) cells. This implies that Ubc9 auto-sumoylation is necessary for extended RLS in *ulp2Δ* 500G (Uba2^C162S^).

Taken together, our results suggest that Ubc9 auto-sumoylation-dependent SUMO target discrimination is a fundamental aspect of lifespan extension in *ulp2Δ* 500G (Uba2^C162S^). This may be connected to changes in energy metabolism, as suggested by our RNA-seq and mitochondrial DNA elimination data, while *ulp2Δ* 500G (Uba2^C162S, A414P^) increases RLS via a distinct route.

### Ubc9 auto-sumoylation affects genome-wide SUMO binding to chromatin.

**Figure 6:**
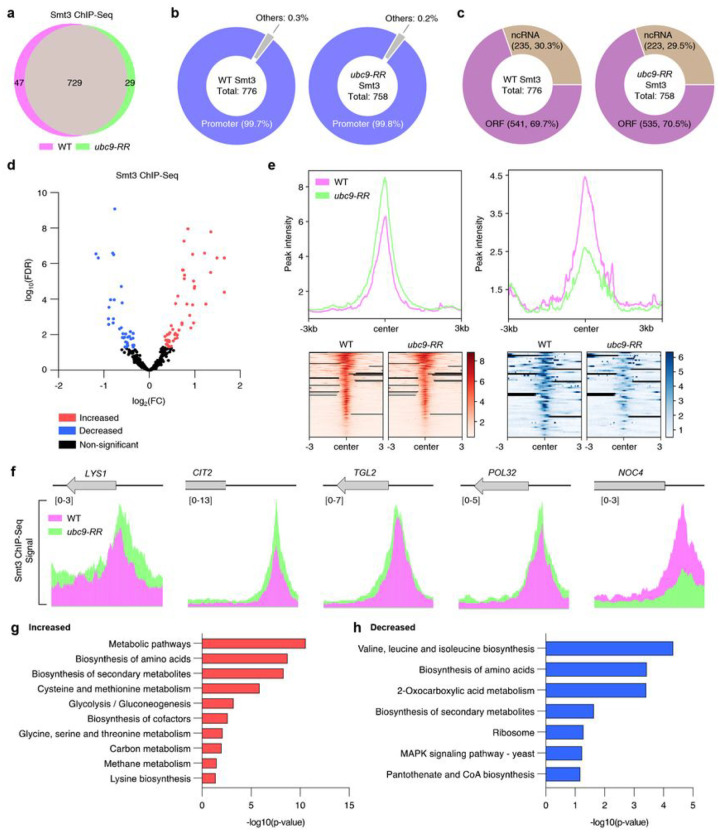
Ubc9 auto-sumoylation-associated changes in the genome-wide pattern of SUMO enrichment. **a**, Venn diagram showing overlapping ChIP-seq peaks of HF-Smt3 (SUMO) in wild-type (WT; magenta) and *ubc9-RR* (green) strains. The ChIP-seq data were obtained from duplicate samples. **b,c**, Pie chart depicting the distribution of HF-Smt3 peaks at promoter and other regions **(b)** and ORF and ncRNA loci **(c)** in **a**. The number in the parentheses indicates the number of identified peaks and their percentages, **d**, Volcano plots displaying the distinct HF-Smt3 peaks in **a**. The y-axis is the mean of the negative logarithm of FDR-corrected P-values. The x-axis corresponds the log2 fold change value. Red and blue dots denote the significantly increased and decreased peaks in *ubc9-RR*, respectively, compared to the WT. **e**, ChIP read-density plot for levels of HF-Smt3 from the significantly increased (left panel) and decreased (right panel) peaks in *ubc9-RR* in d. A ±3 kb window relative to the center of the peaks is shown. Bottom panels indicate heatmaps of SUMO occupancy in WT and *ubc9-RR*. **f**, Representative data in **a**. The y-axis shows fold enrichment normalized to the input DNA. Gray arrows and boxes with gene names indicate locations of ORFs. **g,h**, KEGG analysis of the significantly increased **(g)** and decreased **(h)** peaks displayed in **d**. Bar diagrams indicate the fold-enrichment in each pathway in DAVID (https://david.ncifcrf.gov/).

SUMO has both positive and negative impacts on transcription^48,49^, and its level in chromatin is dynamically regulated by the Ulp2 protease^50^. Transcription factors provide abundant SUMO substrates^39,51^, and SUMO is enriched in numerous loci, primarily the promoter regions of constitutively activated genes and genes involved in translation, such as ribosomal protein and tRNA genes^49,52,53^. Because *ubc9-1* mutation strongly diminishes the association of SUMO with constitutive genes, ribosomal protein genes, and tRNA promoters^49,52^, we examined the role of Ubc9 auto-sumoylation in SUMO occupancy on chromatin via chromatin immunoprecipitation (ChIP)-seq analysis with strains expressing HF-tagged Smt3 (Fig. 6 **and Supplementary Data 3**). The SUMO ChIP-seq data sets contained 776 peaks in the WT and 758 peaks in the *ubc9-RR* mutant. Almost all the SUMO peaks in the two strains overlapped (Fig. 6a). SUMO was almost always located near the promoter regions (99.7% in WT and 99.8% in *ubc9-RR*; Fig. 6b), and of the SUMO-occupied genes, ~ 70% are protein-coding genes and ~ 30% are noncoding RNA gene (Fig. 6c), in agreement with previous results^52,53^. In particular, the *ubc9-RR* mutation contributed both positively and negatively to SUMO localization at diverse gene promoters, including *LYS1, CIT2, TGL2, POL32*, and *NOC4* (Fig. 6d, e, f). KEGG pathway analysis revealed significantly increased and decreased SUMO enrichments in the *ubc9-RR* strain in genes involved in various pathways, including glycolysis, gluconeogenesis, ribosomal translation, and some metabolic pathways (Fig. 6g, h). This finding suggests that Ubc9 auto-sumoylation dynamically regulates chromatin sumoylation at multiple genes related to metabolic and ribosomal pathways.

Similar to the RNA-seq data (Fig. 1b, c), the general chromatin targets of auto-sumoylated Ubc9 were genes involved in energy metabolism and translation. Although SUMO both activates and represses transcription, previous studies suggest that sumoylation is usually associated with transcriptional repression^48,49^. Ubc9 lacking SUMO modifications enhances sumoylation of the glycolysis and gluconeogenesis gene loci, which may result in low levels of their transcripts. By contrast, highly auto-sumoylated Ubc9 in the *ulp2Δ* 500G (Uba2^C162S^) strain correlates strongly with increases in ribosomal gene expression levels, likely via decreased SUMO-chromatin binding at these sites. Thus, the higher ribosomal protein gene transcripts might be attributable to low levels of SUMO at these loci in *ulp2Δ* 500G (Uba2^C162S^).

### Ubc9 auto-sumoylation influences sumoylation of proteins involved in translation and energy metabolism

**Figure 7:**
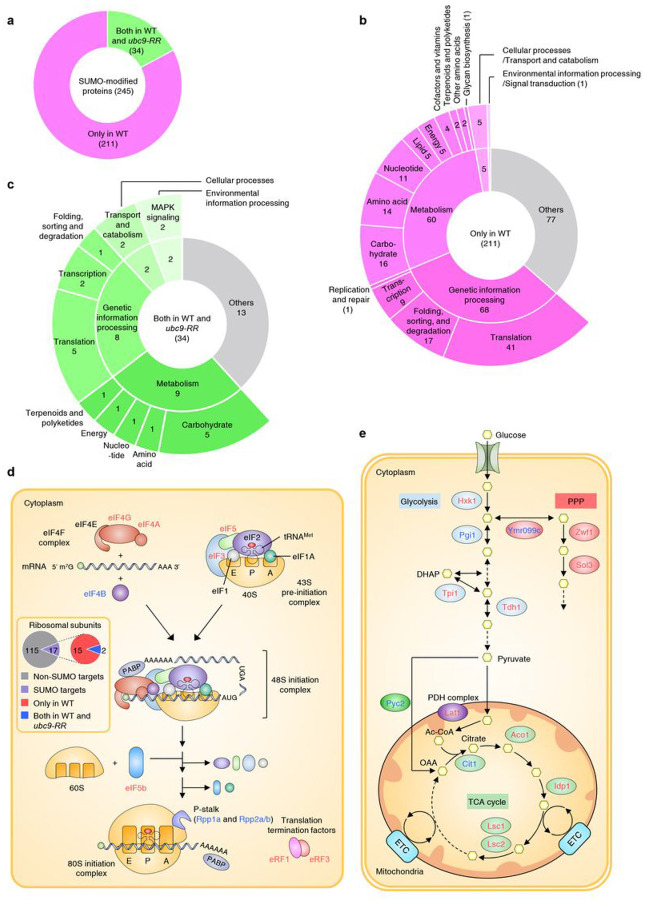
Auto-sumoylation of Ubc9 is required for the regulation of sumoylation of factors involved in translation and energy metabolism. **a**, Pie chart showing the distribution of SUMO-modified proteins in only wild-type (WT; magenta) and in both WT and *ubc9-RR* (green). SUMO-modified proteins were identified via mass spectrometry, as shown in Fig. 4a. **b,c**, KEGG analysis of SUMO-modified proteins in only WT **(b)** and in both WT and *ubc9-RR(c*) displayed in **a**, as shown in Fig. 6g. Numbers indicate the number of proteins identified in each pathway. **d,e**, Schematic diagram showing the list of Ubc9 auto-sumoylation-governed targets in the translation **(d)** and energy metabolism **(e)** pathways. Proteins in the two pathways depicted in **b** and care marked as red and blue colors, respectively. Solid and dashed arrows denote the direction of fluxes and pathways in which intermediate molecules are not depicted, respectively. Metabolites in metabolic reactions are represented as yellow hexagons. The pie graph within **d** shows the distribution of SUMO targets among ribosomal subunits and are classified by the indicated groups. PPP: pentose phosphate pathway; DHAP: dihydroxyacetone phosphate; PDH complex: pyruvate dehydrogenase complex; Ac-CoA: Acetyl-CoA; OAA: oxaloacetate; ETC: electron transport chain.

Ubc9 auto-sumoylation can alter substrate selection by the SUMO-conjugation pathway^22^. To determine whether Ubc9 auto-sumoylation facilitates a broad transition in substrate targeting that might be connected to the genome-wide changes of SUMO-chromatin association (Fig. 6), we compared SUMO-conjugated substrates between the WT and *ubc9-RR* strains expressing HF-Smt3 using affinity purification and mass spectrometry (Fig. 7 and **Supplementary Data 4**). From cells grown in rich media to mid-exponential phase, we identified 245 SUMO-modified proteins only in WT cells, 34 substrates in both the WT and *ubc9-RR* strains, and none that were exclusively seen in *ubc9-RR* cells. This indicated that the number of sumoylated proteins had greatly declined in the auto-sumoylation mutant (Fig. 7a). KEGG enrichment analysis showed that proteins functioning in translation and metabolism constituted the largest classes of proteins that were detected only in the WT sumoylome (Fig. 7b); these categories were also enriched among the sumoylated proteins found in both WT and *ubc9-RR* cells (Fig. 7c).

Translation and metabolism are complex processes. To establish the 80S initiation complex for protein synthesis, the eIF4F complex facilitates mRNA association with the 43S preinitiation complex, which leads to formation of the 48S initiation complex, to which the 60S subunit binds following start codon selection (Fig. 7d)^54^. For the metabolic breakdown of glucose, glycolysis produces pyruvate in the cytoplasm, which is either converted ultimately to ethanol during fermentation or imported into mitochondria for respiration via the TCA cycle (Fig. 7e). SUMO is known to act as a key regulatory factor in both of these pathways^24^. Our data reveal that multiple translation initiation and termination factors, as well as ribosomal proteins, are only detectably sumoylated when Ubc9 can be auto-sumoylated, and the same is true for multiple glycolytic and TCA enzymes. These results suggest Ubc9 auto-sumoylation provides a mechanism to control the sumoylation and presumably levels or activity of proteins directly involved in protein synthesis and energy metabolism.

### Ubc9 auto-sumoylation is increased in aged cells

**Figure 8:**
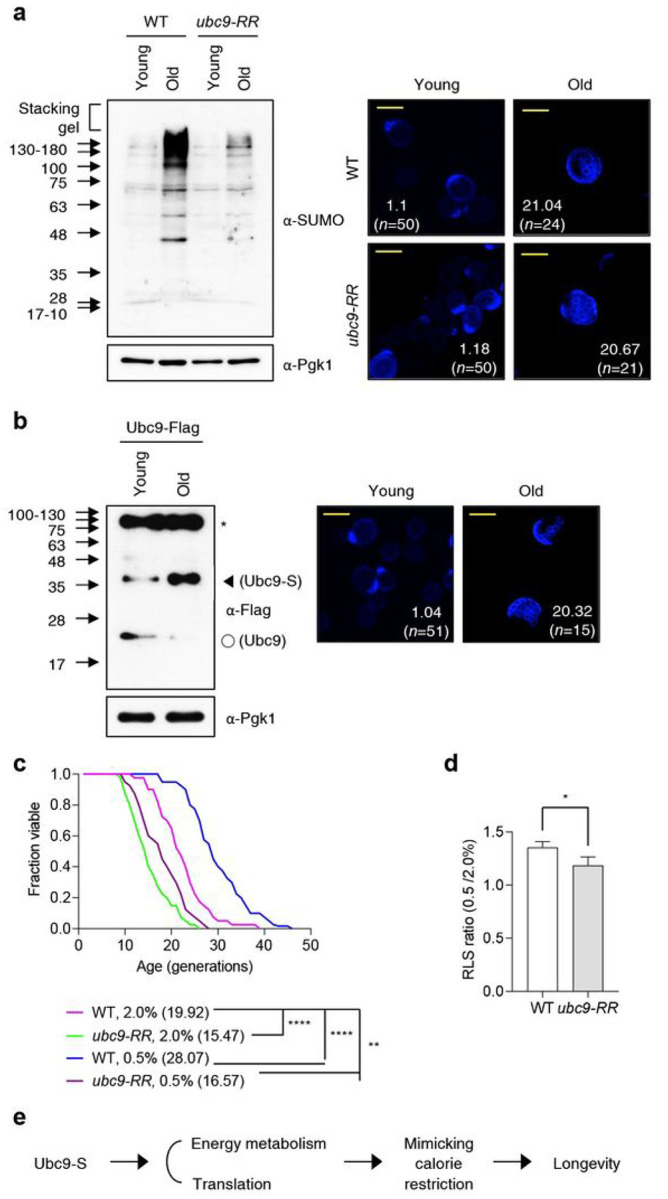
Level of Ubc9 auto-sumoylation is increased in old-aged cells. **a,b**, Immunoblot assay of sumoylated proteins **(a)** and Ubc9-FLAG **(b)** after old cell sorting in the indicated young and old cells, as shown in Fig. 5a. Calcofluor staining images of young and old cells are shown on the right. The number outside of and within the parentheses represents the average number of bud scars (i.e., the replicative age) and the number of cells measured for counting, respectively. Scale bar: 5 μm **c**, RLS analysis of the indicated strains on the SC plates supplemented with 2.0% or 0.5% glucose, as shown in Fig. 3a. **d**, Quantification of RLS ratio of 0.5% to 2.0% in **c** and **Extended Data Fig. 6**. The error bars represent the SD from three independent experiments. Asterisks indicate statistically significant differences (*P< 0.05, two-tailed Student’s t-test). **e**, Schematic diagram depicting the role of Ubc9 auto-sumoylation in longevity.

Aging induces markedly increased sumoylation levels, which contribute to changes in mitochondrial dynamics and mitophagy in *C. elegants*^55^. To look for possible changes in yeast Ubc9 auto-sumoylation in response to aging, we first measured the SUMO conjugate levels in replicatively aged cells (Fig. 8a). Old cells were obtained by isolating biotin-labeled mother cells after three rounds of sorting. We found that the average bud scar number was more than twenty per cell (Fig. 8a, **right panel**). Similar to *C. elegans*, global SUMO conjugates were greatly increased in replicatively aged *S. cerevisiae* cells. By contrast, the *ubc9-RR* mutation blocked much of the age-linked accumulation of SUMO conjugates. This indicates that Ubc9 auto-sumoylation is needed to sustain enhanced sumoylation as yeast age. Notably, Ubc9 auto-sumoylation also increased substantially in aged cells compared to younger cells, and its unconjugated form was almost fully depleted (Fig. 8b). Therefore, it is possible that Ubc9 auto-sumoylation elicits the enhanced SUMO modification of targets in aged cells, and this in turn may promote the survival of aged cells, which we tested next.

### Ubc9 auto-sumoylation is required for RLS extension by CR

In several organisms, CR leads to increased mitochondrial function to boost endogenous energy production and repress ribosome biogenesis and translation via the downregulation of TOR signaling, resulting in a prolonged lifespan^56–59^. Our results (Fig. 1b, c) showed CR-mediated changes in cellular physiological and metabolic characteristics that were also observed in *ulp2Δ* 500G (Uba2^C162S^) that had not undergone CR. Additionally, Ubc9 auto-sumoylation dramatically modulates the sumoylation of proteins in pathways affected by CR (Figs. 6 and 7). To investigate the relationship between Ubc9 auto-sumoylation and CR, we measured RLS in a *ubc9-RR* strain (Fig. 8c). The RLS of WT cells grown on SC plates was slightly lower than those grown on YPD rich medium. As expected from previous work^60^, RLS was clearly extended by reducing glucose content in the media from 2–0.5%. This starvation-induced RLS enhancement was lost in *ubc9-RR* cells. We repeated the RLS analyses three times and concluded that the increased ratio of RLS by lowering glucose concentration from 2.0–0.5% was significantly decreased by the inhibition of Ubc9 auto-sumoylation (Fig. 8d **and Extended Data. 6**). We suggest that Ubc9 auto-sumoylation regulates both transcriptional and post-translational targets of the SUMO pathway, particularly those that are involved in energy metabolism and protein translation to create a longevity-enhancing state that can also be reached through CR (Fig. 8e).

## Discussion

Here we have described the unexpected finding that enhanced Ubc9 auto-sumoylation, a self-regulatory adaptation mechanism caused by the cellular stress response to Ulp2 deficiency, leads to a prolonged lifespan. Ubc9 auto-sumoylation is critical for the discrimination of target proteins and modulates sumoylation of various targets related to energy metabolism and translation at the transcription and post-translational levels. Increased auto-sumoylation contributes to the higher survival of aged cells and prolonged RLS in response to CR. Thus, we demonstrated that SUMO target selection and/or sumoylation activity level likely plays a role as a key regulator of cellular aging by regulating core metabolism and protein synthesis.

Various CR regimens have been shown to increase longevity in different model organisms; this is accompanied by increased mitochondrial activity and numbers, resulting in enhanced ATP production^19^. Although the control of energy metabolism is fundamental to cell growth, decreased growth rate under CR is closely correlated with lifespan extension and slowed aging^61^. Contrary to CR, *ulp2Δ* 500G (Uba2^C162S^) cells display increased energy metabolism and growth rate but also have longer lifespans. This is likely mediated by enhanced Ubc9 auto-sumoylation. These findings suggest that Ubc9 auto-sumoylation promotes higher ATP generation that can enhance replication ability, regardless of the degree of cellular aging.

Although CR can improve the health and lifespan in many organisms, food intake reduction may not be easy to implement or sustain in humans. Thus, many researchers are seeking drugs that can mimic the effects of CR without reducing food intake. These drugs are called calorie restriction mimetics (CRMs) and aim to modulate the same metabolic pathways involved in CR, such as glycolysis inhibition (2-deoxyglucose), enhanced insulin action (metformin), or altered stress signaling pathways (resveratrol)^62–64^ While most CRMs have focused on limiting or activating a specific step in the metabolic pathway that results in the restriction of energy source utilization, Ubc9 auto-sumoylation control can simultaneously affect multiple targets linked to various pathways and can facilitate nutrient consumption. Because the antiaging effects of CR involve diverse pathways, interventions intended to provide control to multiple systems could be more beneficial. Thus, the maintenance or enhancement of Ubc9 auto-sumoylation could potentially offer a novel way to limit aging and age-related diseases.

## Methods

### Yeast strains and plasmids

Yeast strains and plasmids used in this study are listed in **Supplementary Tables 1 and 2**. Standard techniques were used for strain construction. To generate HYS332, HYS343, HYS452, HYS453, HYS454, and HYS592 strains, the C-terminal tagging cassette from pFA6a-6xGly-3xFlag::KanMX6 plasmid^65^ was amplified using PCR and inserted into the *UBC9* locus in HYS69, HYS72, HYS88, HYS114, and HYS216 strains. For the construction of *6xHis-Flag-SMT3* strains, the N-terminal tagging cassette was amplified from HYS40 strain^66^. The *sir2Δ* strains were generated by replacing *SIR2* ORF in HYS88, HYS567, and HYS568 strains via *kanMX4* modules constructed using PCR amplification from HYS209 obtained from Euroscarf. To make HYS575, the *HIS3MX6* marker in HYS568 was swapped with the *TRP1* marker via the transformation of *Not*I-digested M4757 (*kanMX::TRP1* converter plasmid^67^). The *ubc9-RR* strains were generated by replacing *UBC9* ORF with the *ubc9-K153/157R::HIS3MX6::TRP1* cassette from HYS575. The genomic tiling library plasmids containing the appropriate genes were transformed into the HYS88 strain to overexpress the genes of interest. To create yeast strains lacking *ULP2, ulp2Δ + YCplac33-ULP2* haploid strains (HYS79, HYS88, HYS126, HYS332, HYS576, HYS596, and HYS604) with or without a *LEU2*-marked plasmid carrying a gene(s) of interest were twice streaked on 5-FOA plates with or without leucine to evict the *ULP2* cover plasmid. The rho^0^ strains were generated by growth in YPD medium supplemented with ethidium bromide (25 μg/mL)^68^. All strains were verified using PCR and/or immunoblot analysis.

### Yeast growth conditions

Cells were grown at 30°C in YPD or synthetic defined (SD) medium using appropriate supplements. Cultures in exponential growth were normalized to an OD_600_ of 0.1 for growth analysis on plates and were subject to fivefold serial dilutions. Dilution series were spotted onto the appropriate media, and the plates were incubated at 30°C and 37°C for 1 to 3 days. To measure the growth rates in liquid cultures, we diluted overnight cultures to an OD_600_ of 0.1 and then measured the cell density with a spectrophotometer every 6 h. During the *in vitro* laboratory evolution experiments, the cells were grown to stationary phase in YPD, then diluted at a ratio of 1:120 into fresh YPD medium, as previously described^69^. This process was repeated daily until 500 cell generations were reached (i.e., at a rate of 6.9 generations per day).

### RLS analysis

The RLS of the yeast strains were measured on YPD plates as previously described^70^ unless otherwise indicated. A total of about 50 virgin daughter cells were subjected to RLS analysis using a microscope equipped with a micro-dissection apparatus (SporePlay, Singer Instruments). The Mann-Whitney test was performed with a cutoff of P = 0.05 to assess lifespan differences. The average lifespan was considered significantly different when *P*< 0.05.

### RNA-seq

One OD_600_ equivalent of exponentially growing cells in YPD medium were used for RNA isolation using the RNeasy Mini Kit (74104, Qiagen). Contaminating DNA was removed from the samples via the DNA-free^™^ DNA Removal Kit (AM1906, Ambion).

The RNA-seq libraries were constructed according to the Yale Center for Genome Analysis Guidelines. The libraries underwent 101 bp paired-end sequencing using an Illumina HiSeq 2500. The low-quality reads were trimmed and adaptor contamination was removed using Trim Galore (v0.5.0). The trimmed reads were mapped to the *S. cerevisiae* genome (sacCer3) using HISAT2 (v2.1.0)^71^. Gene expression levels were quantified using StringTie (v 1.3.3b)^72^ with gene models from SGD^34,35^. Differentially expressed genes were identified using DESeq2 (v 1.22.1)^73^.

### DNA-seq

Genomic DNA was extracted from 1 OD_600_ of cells as previously described^14^. Whole-genome sequencing was then performed according to the Yale Center for Genome Analysis Guidelines, using 2 × 100 bp sequencing on an Illumina HiSeq 2500 to an average depth of 500× per sample. The reads were aligned to the *S. cerevisiae* genome using BWA MEM v0.7.15, and a joint calling of variants was performed using FreeBayes v0.9.14 with the following options: -P 0.5; -E 0; -q 10; -C 5.

### ChIP-seq

ChIP experiments were performed as previously described^74^. Briefly, formaldehyde was added to mid-log phase cells at a final concentration of 1% for 20 min. Cross-linking was quenched by adding 240 mM glycine. The cross-linked cells were collected via centrifugation, washed twice in ice-cold Tris-buffered saline (TBS), then lysed by ten 30 s pulses of vortex mixing with glass beads in an FA lysis buffer (50 mM HEPES–KOH; pH 7.5; 150 mM NaCl; 1 mM EDTA; 1% Triton X-100; 0.1% Na deoxycholate; 0.1% SDS; protease inhibitor cocktail (4693132001, Roche), 1 mM PMSF (11359061001, Roche)). Chromatin sheared via sonication was incubated with anti-Flag M2 agarose beads (F3165, Sigma-Aldrich) at 4°C for 14 h. The precipitates were then sequentially washed with FA lysis buffer and 275 mM NaCl, FA lysis buffer with 500 mM NaCl, LiCl washing buffer (10 mM Tris–HCl; pH 8.0; 0.1 mM EDTA; 250 mM LiCl; 0.5% NP-40; 0.5% sodium deoxycholate), and TE (10 mM Tris–HCl; pH 8.0; 1 mM EDTA), then eluted with elution buffer (10 mM Tris–HCl; pH 7.5; 10 mM EDTA; 1% SDS) at 65°C. Eluted chromatin fragments were treated with protease (P5147, Sigma-Aldrich), then DNA was extracted using a standard phenol/chloroform extraction method.

The ChIP-seq libraries were constructed using a TruSeq DNA Sample prep kit and sequenced on an Illumina NovaSeq 6000 according to the manufacturer’s protocols (TruSeq ChIP Sample preparation guide 15023092 Rev. B). Reads were trimmed using a Trimmomatic (v0.38) and aligned to the yeast reference genome (sacCer3) using BowTie (v1.1.2). Peaks were called utilizing MACS (v2.1.1.20160309) and duplicate reads were processed using Picard (v0.118). Called peaks were annotated using ChIPSeeker (v1.16.1) with gene models from SGD (https://www.yeastgenome.org/). Comparative data analyses were performed using csaw (v1.34.0). The normalized bedGraph files were generated using MACS2 (‘-B −SPMR’), then converted to bigWig files using the bedGraphToBigWig program. A genome-wide profile was generated using the ‘computeMatrix scale-regions’ and plotProfile tools of the deepTools package (v3.1.3).

### Immunoblotting

Preparation of yeast whole-cell extracts and immunoblotting preparations were carried out as described previously^75^. The levels of FLAG-tagged Ubc9, SUMO-conjugate profiles, and Pgk1 were analyzed via immunoblot assay using anti-FLAG (F3165, Sigma-Aldrich), anti-SUMO (Mark Hochstrasser Laboratory^76^ and ab14405, Abcam), and anti-Pgk1 (459250, Molecular Probes), respectively. sumoylated Ubc9 was detected as previously described with little modification^14,77^. Briefly, TCA-extracted proteins from 50 OD_600_ cells were resuspended in 0.6 mL 1× Laemmli sample buffer (60 mM Tris–HCl; pH 6.8; 2% SDS; 10% Glycerol; 5% (B-mercaptoethanol; 0.008% Bromophenol blue) with 100 μ**L** unbuffered 2 M Tris, then heated at 100°C for 5 min. The solution was centrifuged twice at 15,000 rpm for 10 min, and then the protein concentration of the supernatant was determined via a Bradford protein assay. Next, 2 mg of the protein was incubated with prewashed 40 μL anti-FLAG M2 agarose beads (A2220, Sigma-Aldrich) in IP buffer (50 mM Tris; pH 7.4; 150 mM NaCl; 0.5% NP-40; 20 mM NEM (E3876, Sigma-Aldrich)) at 4°C for 4 h. After washing three times with IP buffer, the bound protein was eluted with 40 μL of 5× Laemmli sample buffer by heating at 100°C for 5 min. Both the IP and INPUT samples were analyzed via immunoblot assays as described above.

### Tandem affinity purification of sumoylated proteins and mass spectrometry

Purification of sumoylated proteins was performed as previously described with little modification^66^. Cells expressing 6xHis-FLAG-tagged Smt3 were grown to OD_600_ 1.0 in 4 L YPD and harvested via centrifugation at 4,000 rpm for 10 min. The cell pellets were lysed by ten 30 s pulses of vortex mixing with glass beads in a denatured lysis buffer (100 mM NaH_2_P0_4_; 10 mM Tris–HCl; pH 8.0; 0.1 % SDS; 8 M urea; protease inhibitor cocktail (4693132001, Roche), 1 mM PMSF (11359061001, Roche)). Cell debris were removed by centrifuging twice at 18,000 rpm for 30 min each. Protein concentration was measured using the Bradford protein assay, and then 50 mg of the proteins were incubated with 2 mL of prewashed Ni-NTA agarose resin (R90115, Invitrogen) at 4°C for 4 h and loaded into a column. The resin was sequentially washed with 20 mL denatured wash buffer (100 mM NaH_2_PO_4_; 10 mM Tris−HCl; pH 8.0; 6 M urea), cold native wash buffer (50 mM NaH_2_P0_4_; 10 mM Tris–HCl; pH 8.0; 150 mM NaCl; 0.02% Tween 20; 20 mM imidazole) with 4 M urea, cold native wash buffer with 2 M urea, and cold native wash buffer. Proteins were eluted with elution buffer (50 mM NaH_2_P0_4_; 10 mM Tris–HCl; pH 8.0; 150 mM NaCl; 0.02% Tween 20; 300 mM imidazole) and then incubated with 200 μL anti-FLAG M2 agarose beads at 4°C for 4 h. The beads were washed three times with cold native wash buffer without imidazole, then once with cold water to remove the salts. Finally, the sumoylated proteins were eluted using 0.1 M glycine (pH 2.5) and were concentrated via an Amicon Ultra-4 centrifugal filter (UFC803008, Millipore).

Identification of sumoylated proteins in *ulp2Δ* 500G (Uba2^C162S^) and *ulp2Δ* 500G (Uba2^C162S^) cells was performed using a Q-Exactive plus spectrometer (ThermoFisher) following the Keck MS & Proteomics Resource Guidelines. Proteins were reduced with DTT and alkylated with MMTS prior to extraction using a cold acetone protein precipitation procedure. Precipitated proteins were dissolved in an acid labile detergent (Rapigest^™^, Waters Inc.) at a 0.1*%* concentration in 50 mM NH_4_HCO_3_. 20 μL of a 1:5 dilution of stock trypsin (1 μg/μL; Promega) was added to the protein mixture, and digestion was carried out with 4 μl of 0.1 mg/ml trypsin (Promega) was added, and the samples were digested at 37°C for 16 h. The digest was quenched, and Rapigest^™^ was removed by the addition of 20% trifluoracetic acid at 37°C for 45 min. Samples were stored at −20°C until just prior to analysis by liquid chromatography-tandem mass spectrometry (LC-MS/MS). LC-MS/MS data were analyzed utilizing Proteome Discoverer 2.4 (Thermo Fisher Scientific) with Mascot search engine (v. 2.7 Matrix Science LLC) using the *Saccharomyces cerevisiae* protein database. The resulting PD analyses were imported into Scaffold (v. 4.1, Proteome Software) for further interrogation and data filtration. Positive protein identifications were based on hits with two or more unique peptides per protein, and peptides were considered significant if the Mascot Score is better than the 95% confidence level. The protein database searched in-house using the Mascot algorithm (v. 2.2.0) for un-interpreted MS/MS spectra. The data was searched against the SWISSPROT *Saccharomyces cerevisiae* protein database.

Sumoylated proteins in *ubc9-RR were* identified using a Q-Exactive plus spectrometer (ThermoFisher) through a nano-electrospray ion source. LC-MS/MS identification of the purified sumoylated proteins were conducted at the New Drug Development Center, KBIO, Osong, Korea. Following the same general procedures as previously described^78^,100 μg of purified protein samples were reduced, alkylated, digested with Trypsin/Lys-C mix (V5073, Promega), desalted using OASIS SPE Cartridge (1860000383, Waters), then dried before being run. The dried peptides were examined via online nanoflow LC-MS/MS using a UPLC system (Waters, Millford), which was connected to a Q-Exactive spectrometer through a nano-electrospray ion source to gather particular MS/MS spectra. Proteome Discoverer (SEQUEST, Thermo Fischer Scientific, ver. 1.4.0.288) and Scaffold (X! Tandem, version Scaffold_4.4.6, Proteome Software, Portland, OR) were used to search the databases. The MS/MS data was analyzed against an *S. cerevisiae* database (S288C). Low-quality identified proteins were removed when they fell below the unique peptide score 1, number of peptides 1, and coverage of 7%. The KEGG pathway database (https://www.genome.jp/kegg/pathway.html) was used for functional classification of the identified SUMO target proteins.

### Isolation of aged yeast mother cells

The aged mother cells were isolated as previously described with some modifications^79^. Thirty OD_600_ equivalents of cells grown to exponential phase in YPD were harvested, then labeled with 24 mg of EZ-Link^™^ Sulfo-NHS-LC-Biotin (21335, Thermo Fisher) in 1 mL phosphate-buffered saline (PBS) followed by gentle rotation at room temperature for 20 min. Cells were then washed with 1 mL PBS containing 0.1 M Glycine three times, and the biotin-labeled cells were resuspended in 1 L YPD and incubated at 30°C for 12–14 h. For the first round of sorting, the harvested cells were incubated with Dynabeads^™^ Biotin Binder (11407, Thermo Fisher) at 4°C for 1 h. The biotin-labeled cells were collected with a DynaMag^™^-15 magnet (12301D, Thermo Fisher) and subsequently washed 9 times with 10 mL cold PBS. Older cells obtained from the second and third rounds of sorting were also inoculated into YPD at 10^8^ cells per 1 L and grown at 30°C for 12–14 h. The sorting procedure was then repeated and about 2 × 10^5^ aged cells were saved for immunoblot analysis as described above. To estimate the average number of bud scars, which indicates the number of generations, cells were stained with 10 μM of Calcofluor White M2R (Sigma-Aldrich, 910090), and then the bud scar numbers were counted in composite images created using an IX83 confocal microscope (Olympus).

## Data availability

Data generated or used in this publication have been deposited in Gene Expression Omnibus (GEO) and Proteomics Identifications Database (PRIDE) with accession numbers:

- RNA-seq data: GSE254981
- ChIP-seq data: GSE253055
- Proteome data: PXD048944 (username: reviewer_pxd048944@ebi.ac.uk; password: ci7d2qAM) and PXD049427 (username: reviewer_pxd049427@ebi.ac.uk; password: XjboGzhs)
